# Experiencing Physical Pain Leads to More Sympathetic Moral Judgments

**DOI:** 10.1371/journal.pone.0140580

**Published:** 2015-10-14

**Authors:** Qianguo Xiao, Yi Zhu, Wen-bo Luo

**Affiliations:** 1 College of Education and Science, Inner Mongolia Normal University, Hohhot, China; 2 Laboratory of Cognition and Mental Health, Chongqing University of Arts and Sciences, Chongqing, China; 3 International Business School, Southwestern University of Finance and Economics, Chengdu, China; 4 School of Psychology, Liaoning Normal University, Dalian, China; University of Bologna, ITALY

## Abstract

Previous studies have shown that observing another’s pain can evoke other-oriented emotions, which instigate empathic concern for another’s needs. It is not clear whether experiencing first-hand physical pain may also evoke other-oriented emotion and thus influence people’s moral judgment. Based on the embodied simulation literature and neuroimaging evidence, the present research tested the idea that participants who experienced physical pain would be more sympathetic in their moral judgments. Study 1 showed that ice-induced physical pain facilitated higher self-assessments of empathy, which motivated participants to be more sympathetic in their moral judgments. Study 2 confirmed findings in study 1 and also showed that State Perspective Taking subscale of the State Empathy Scale mediated the effects of physical pain on moral judgment. These results provide support for embodied view of morality and for the view that pain can serve a positive psychosocial function.

## Introduction

Pain is defined as “an unpleasant sensory and emotional experience arising from actual or potential tissue damage or described in terms of such damage” [1].Traditionally, scholars tended to regard pain as a form of negative emotion, associated it with such phenomena as harm and illness. Recent studies suggested that pain can also have positive psychosocial functions: Perception of pain can evoke other-oriented affection [2], motivate people to help others, promote interpersonal trust, and increase cooperation in public goods game [3] and acceptance rate in an ultimatum game [4]. Drawing on evidence from a range of academic fields, Bastian et al. [5] concluded that pain has positive psychosocial functions in terms of facilitating pleasure, augmenting self-regulation, and enhancing interpersonal connection.

Among these positive functions of pain, empathy plays an important role in influencing observers’ helping behaviors [6]. The empathy-altruism hypothesis has been supported by previous studies [7–9]. With respect to the neurological mechanisms underlying empathy, several neuroimaging studies showed that paying attention to others in pain activated neural structures that overlap those involved in the direct experience of pain, including the anterior insula (AI) and anterior cingulate cortex (ACC), adjacent inferior frontal cortex [10–12], resulting in shared representations between self and target persons [13]. Recently, numbers of researches showed that empathy is often connected with broader neural regions including the brainstem, amygdala, hypothalamus, striatum, insula, anterior cingulate cortex, orbitofrontal cortex, parasympathetic and sympathetic branches [14–16], as well as being associated to somatosensory cortex [17–21]. These neuroimaging findings implied that experiencing personal pain could also activate relevant neural substrates, facilitating people’s understanding of others’ pain. However, some other findings suggested that indirect pain representations (as elicited by the observation of pain in others) and the actual experiences of pain can be qualitatively different [11,22].

Though it is far from clear whether experiencing first-hand physical pain would instigate empathic concern for others, embodied simulation studies suggest that empathy for others’ pain hinges largely on an observer’s own emotional and sensory experiences [23,24]. Neuroscientists believe internal simulation to be the mechanism underlying linkages between one’s own personal feelings and another’s experiences, and mirror neuron systems are believed to be the basis of such shared representation [25–27]. A number of studies provided evidence for the role of embodied simulation in shaping empathy. For instance, the ability to understand pain or distress in others was found to rely on personal body-related experiences [28], particularly prior pain experiences which lead to more readily elicited empathic responses [29,30]. Recent evidence showed that sensorimotor cortex is activated in the embodied condition (such as touch) and is associated with the empathy [31,32].These evidence is believed to be associated with a pattern of activity in brain embodying observers’ own states [33].

To summarize, prior work suggests that feelings of physical pain, empathy, and prosocial behaviors could be positively correlated, and that first-hand pain could be one important basis of helping people understand others in pain, thus affecting people’s judgment of another’s behaviors.

Although empathy for people in pain has been widely studied, to our best knowledge, few studies have addressed the role of pain-evoked empathy in moral judgment. Based on the aforementioned evidence, we argued that first-hand experience of physical pain facilitates people’s understanding of similar experience of others. Therefore, we predicted that participants who experienced physical pain would be more sympathetic in judging moral dilemmas, and that empathy would mediate the effect of physical pain on moral judgment.

## Study 1

### Materials and methods

#### Participants

One hundred and thirty-one undergraduate students (67 males, 64 females; *M*_age_ = 19.26 years, *SD* = 1.16) at a Chinese university voluntarily participated in this experiment. Each participant was paid 5 RMB for participation. The study employed a 2 (pain manipulation: pain vs. no pain) x 2 (moral scenario: pain cue vs. no pain cue) between-subjects design. Participants were randomly assigned to one of four conditions—66 in the pain group (13 girls and 21 boys in the pain cue condition, *M*_age_ = 19.05; 16 girls and 16 boys in the no pain cue condition, *M*_age_ = 19.44), and 65 in the no pain group (19 boys and 15 girls in the pain cue condition, *M*_age_ = 19.88; 16 boys and 15 girls in the no pain cue condition, *M*_age_ = 18.74). Participants were informed that the study was about temperature perception and were given their written consent after they understood the protocol and agreed to participate in the experiment. The experiment was approved by the Chongqing University of Arts and Sciences institutional review board and ethics committee.

### Procedure and measures

#### Pain manipulation

Following Bastian et al. [34], participants in the pain condition were instructed to immerse their non-dominant hand up to the wrist in a bucket of icy water (0°C-2°C) as long as possible, while participants in the no pain condition were instructed to immerse their non-dominant hand in a bucket of warm water (approximately 30°C) for 90 seconds.

To check the effectiveness of our physical pain manipulation, a numerical pain rating scale (NRS-10) was used to measure participants’ feelings of physical pain after the manipulation. The NRS-10 was developed based on a scale originally developed by Scott and colleges [35]. Participants were instructed to indicate the number that best represents their feelings of pain from 0 to 10 (0 represents no pain, 5 represents moderate pain, and 10 represents extreme pain). Empirical studies have demonstrated that the NRS-10 has good sensitivity and generates useful data for clinical applications and research [36,37].

#### Measures

Immediately after the pain manipulation, participants were instructed to complete a package of questionnaires to measure their emotions, empathy, and moral judgment.

***Emotion measure***: The Chinese version of the Positive and Negative Affect Scale (C- PANAS) was used to measure positive and negative emotions in our experiment. The C-PANAS was revised by Qiu, Zheng [38] based on the original PANAS developed by Clark Watson [39]. The C-PANAS has been validated as a good measure of self-reported affective experience among Chinese individuals.

***Empathy measure***: The Interpersonal Reactivity Index (IRI) is a well-validated measure of dispositional empathy. The Chinese version of the Interpersonal Reactivity Index (C-IRI) is designed to measure participants’ degree of empathetic concern. The C-IRI was revised by Rong, Sun [40] based on the original IRI developed by Davis [41]. The C-IRI involves four subscales, Fantasy (FS), Empathic Concern (EC), Perspective Taking (PT), and Personal Distress (PD). Following existing study [42], we only used the EC, PT, and PD subscales to assess participants’ pain-evoked empathy. EC measures emotional aspect of empathy as it relates to the affective experience of feeling compassion for others’ misfortune (sample item: “When seeing those who are hurt or struggling, I really want to help them.”); PT assesses the cognitive component of empathy as it relates to the cognitive ability to comprehend another’s point of view (sample item: “I try to look at everybody’s side of a disagreement before I make a decision.”); PD measures the degree to which one feels anxiety or discomfort viewing others’ anguish (sample item: “When I see someone who badly needs help in an emergency, I go to pieces.”). C-IRI items are rated on a 5-point Likert scale, and there are seven items for each subscale. The construct reliability of C-IRI subscales ranges from .59–.75, and test-retest reliability of the subscales ranges from .59–.78. Table 1 shows the descriptive statistics of the three subscales of C-IRI used in this study. There was no negative inter-correlation between these subscales in the present sample. Following existing studies [43–45], a total score of the three subscales was used as an indicator of trait empathy.

**Table 1:** Summary of descriptive statistics and correlation analysis results.

	*M*	*SD*	1	2	3	4	5	6
1.Moral judgment	3.18	.98	1					
2. C-IRI (Empathic concern)	25.62	3.89	.16	1				
3. C-IRI (Perspective taking)	21.64	2.55	.18*	.28**	1			
4. C-IRI (Personal distress)	20.74	3.66	.11	.15	.105	1		
5. C-IRI (Total scores)	68.00	6.85	.21*	.76**	.59**	.66**	1	
6. Physical pain	3.52	3.06	.27**	.12	.24**	.11	.22*	1

*. Correlation is significant at the 0.05 level (2-tailed).

**. Correlation is significant at the 0.01 level (2-tailed).

NOTE. *N* = 128. Correlation between physical pain, the total scores of C-IRI (three factors), and moral judgment.

#### Moral scenarios

All participants were instructed to read one of two versions of a moral dilemma. The moral dilemmas included in the questionnaire package were “Heinz Steals the Drug” and its revised version. The “Heinz Steals the Drug” story involves two moral stances: one endorses Heinz who steals drugs to save his ill wife, with such stance representing empathy-orientated compassion; the other does not endorse Heinz’ conduct and it represents law-orientated fairness.

Moreover, studies indicate that empathic caring can result from participants’ attentional bias toward the whole state of pain or distress, rather than specific pain cues [28]. Thus, we included a manipulation of such information (pain cue vs. no pain cue) in moral dilemmas, aiming at disentangling whether the effect of physical pain on moral judgment is due to attentional bias toward specific pain cues or the whole “in need of help” state in such scenarios. More specifically, in the revised version of the “Heinz Steals the Drug” story, we controlled the pain cue by changing the description “a woman suffering from a special kind of cancer, involving great pain” into “a woman suffering a strange illnesses without feelings of pain but involving death in the near future”(see brackets in the moral scenario below). After reading the moral scenarios, participants judged to what extent Heinz’s conduct was moral or immoral by responding on a 5-point Likert scale, where 1 represents completely immoral, and 5 represents completely moral. Participants’ responses to the two moral dilemmas were averaged as an index of their “moral judgment”.

"In Europe, a woman was suffering from a special kind of cancer, with great pain (suffering from a strange illness, without any pain, but involving death in the near future).There was one drug that the doctors thought might save her. It was a form of radium that a druggist in the same town had recently discovered. The drug was expensive to make, but the druggist was charging ten times what the drug cost him to make. He paid $200 for the radium and charged $2,000 for a small dose of the drug. The sick woman's husband, Heinz, went to everyone he knew to borrow the money, but he could only get together about $ 1,000, which is half of what the drug cost. He told the druggist that his wife was in great pain and asked him to sell it cheaper or to let him pay later. But the druggist said: "No, I discovered the drug and I'm going to make money from it." So Heinz got desperate and broke into the man's store to steal the drug for his wife. How moral or immoral was it for Heinz to steal the pharmacist’s drug?"

### Results

#### Manipulation check

Three participants were excluded from data analysis because they did not follow research assistant’s instruction to fully submerge their hand in the ice bucket. We first compared pain ratings between the control (*n* = 65) and pain (*n* = 63) conditions. An independent sample *t*-test showed that the pain ratings were significantly different across the two conditions (*t* (126) = 19.94, *p* < .001; pain: *M* = 6.22, *SD* = 1.72; control: *M* = .91, *SD* = 1.27). This analysis showed that our pain manipulation was a valid means to induce genuine bodily pain. We also employed the PANAS to measure participants’ emotional arousal after the physical pain manipulation. The difference in terms of emotional arousal between pain and no pain conditions was not significant (for the PA subscale, *t* (126) = 1.47, *p* > .05, pain: *M* = 2.58, *SD* = .86; control: *M* = 2.35, *SD* = .92; for the NA subscale, *t*(126) = .93, *p* > .05, pain: *M* = 1.33, *SD* = .42; control: *M* = 1.263, *SD* = .41), thus ruling out the possibility of the effects of positive/negative emotion on participants’ moral judgments.

#### Hypothesis testing

Descriptive statistics and correlation analysis results are shown in Tables 1 and 2. Results of an independent sample *t*-test showed that empathy score in the pain condition (*M* = 69, *SD* = 6.55) was significantly higher than in the no pain condition (*M* = 66, *SD* = 6.92), *t* (126) = 2.25, *p* < .05, indicating that feeling physical pain enhanced participants’ self-assessments of empathy.

**Table 2:** Means and Std. Deviation of the three subscales of C-IRI.

Moral scenarios	Empathic Concern	Perspective Taking	Personal Distress
Pain with pain cues	25.97±3.40	22.50±2.25	21.26±4.18
Pain with no pain cues	26.31±3.53	22.06±2.37	20.55±4.05
No Pain with pain cues	24.52±4.71	21.14±2.57	20.05±2.98
No Pain with no pain cues	25.77±3.63	20.83±2.73	21.09±3.35

**Fig 1:**
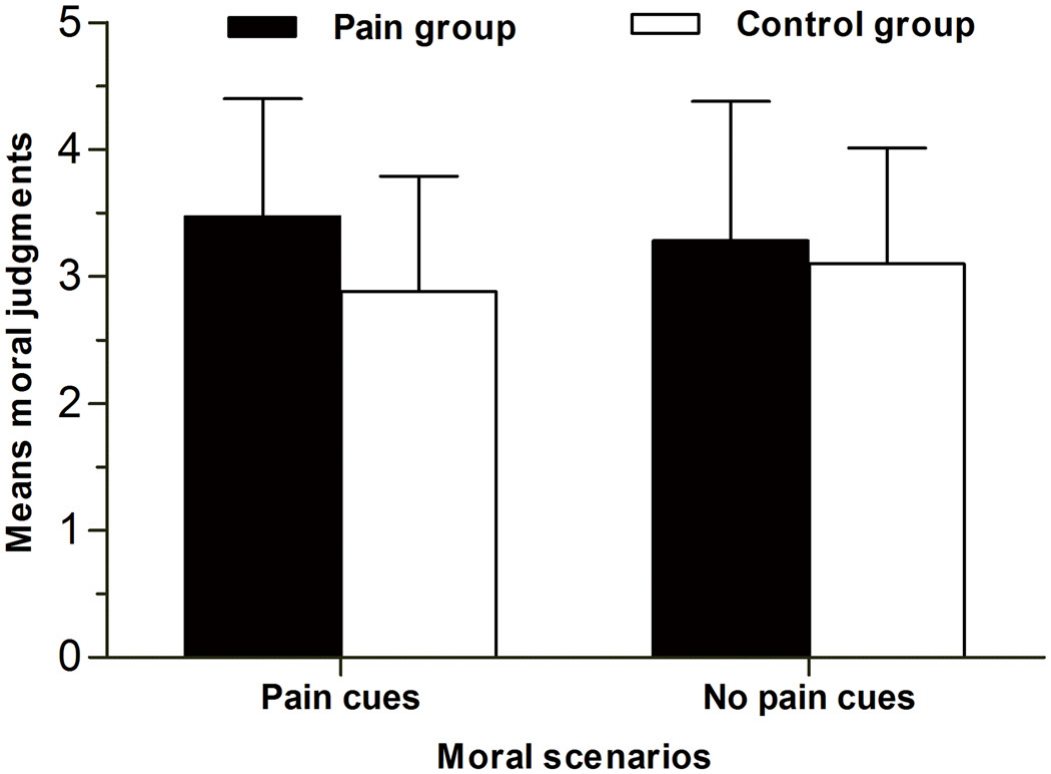
The bar graph of moral judgment in study one. This bar graph provides comparative view of the moral judgment across experimental conditions.

An analysis of variance (ANOVA) on moral judgment revealed a main effect of pain on moral judgment, *F* (1, 124) = 5.08, *p* = .03, *η*_p_^2^ = .04, suggesting that participants in the pain condition were more tolerant of Heinz’s behavior than those in the no pain condition (see Fig 1). However, we did not observe a significant main effect of moral scenario, *F* (1, 124) = .003, *p* > .05, *η*_p_^2^ = .00, nor did we observe a significant interaction between pain manipulation and moral scenario, *F* (1, 124) = 1.44, *p* = .23, *η*_p_^2^ = .01.

**Fig 2:**
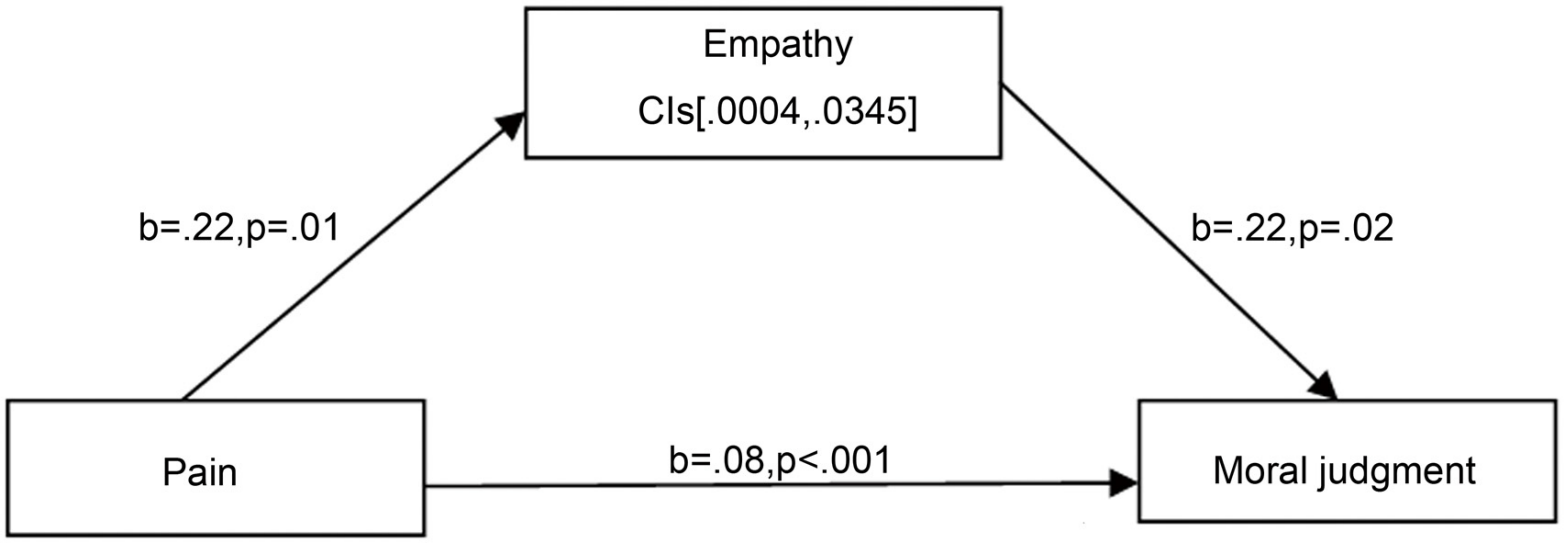
The mediation path of the effect of physical pain on moral judgment. Path model shows that the effect of physical pain on moral judgment is partially mediated by empathy. Bias-corrected and accelerated 95% confidence intervals(CIS) from 5,000 bootstrap samples are reported for the indirect effect.

In addition, we found that the pain, empathy, and moral judgments were significantly correlated with one another (see Table 1). Combined with the above hypothesis, this finding implied that the impact of pain on moral judgment might be mediated by empathy. Thus, we performed a mediation analysis following the bootstrapping procedures outlined by Preacher and Hayes [46]. The results showed that pain had a statistically significant effect on empathy (*b* = .22, SE = .20, *p* = .01), which, in turn, significantly affected moral judgment (*b* = .22, SE = .01, *p* = .02). The bootstrap analysis (with 5,000 iterations) showed that the 95% bias-corrected confidence interval for the size of the indirect effect excluded zero [.0004, .0345], indicating that the indirect effect through empathy was significant. Moreover, the direct effect of physical pain on moral judgment was non-significantly reduced (b = .08, SE = .02, p < .001) (see Fig 2). We also performed mediation analysis separately considering each C-IRI subscale, but unfortunately we did not get the separate mediation effect.

## Study 2

Study 1 shows that ice-induced physical pain facilitated self-assessments of empathy, which motivated participants to be more sympathetic in their moral judgments. This finding also suggested that empathy invoked by seeing others’ pain can be modulated by trait-differences in empathy. In Study 1, we randomly assigned participants to the four experimental conditions, however, we could not rule out the possibility that participants that were assigned to the physical pain condition happened to be higher in trait empathy. In order to control for individual differences in trait empathy, we conducted a second study in which we measured participants’ trait empathy and state empathy respectively before and after experimental manipulations.

### Materials and methods

#### Participants

Undergraduate students (*N* = 120, 47 men and 73 women; *M*_age_ = 20.03 years, *SD* = 1.46) at a Chinese university voluntarily participated in this experiment. Each participant was paid 5 RMB (approximately US$ 0.81) for taking part in the study. This study used a 2 (pain manipulation: pain vs. no pain) x 2 (moral scenario: pain cue vs. no pain cue) between-subjects design. Participants were randomly assigned to one of the four conditions. There were 60 participants in the pain group (14 men and 16 women in the pain cue condition, *M*_age_ = 20.23 years; 9 men and 21 women in the no pain cue condition, *M*_age_ = 20.13 years), and 60 participants in the no pain group (12 men and 18 women in the pain cue condition, *M*_age_ = 20.16 years; 12 men and 18 women in the no pain cue condition, *M*_age_ = 19.66 years). Participants were informed that the study was about temperature perception. All participants gave their written consent to participate in the study, after the protocol of the study was explained to them.

### Procedure and measures

Pain manipulation and moral scenarios used in this study were identical to those used in the Study 1. In Study 2, participants also responded to trait empathy and state empathy scale.

***Trait empathy measure*:** Before the physical pain manipulation, all participants were required to respond to the Basic Empathy Scale (BES), which was used to measure their propensity for trait empathy. This scale was revised by Xia [47] based on the original EBS developed by Jolliffe et al. [48]. The BES assesses two dimensions: cognitive empathy (CE) and affective empathy (AE). The Cronbach’s alphas of the BES are .72 (CE) and .73 (AE), and that the test-retest reliability of the subscales are .60 (CE) and .71 (AE).

***State empathy measure*:** Immediately after the pain manipulation, participants were instructed to complete a state empathy scale. We reasoned that a measure of state empathy might capture participants’ empathic reactions toward situational cues better [49]. Thus, following Trobst et al. [42], we developed a state empathy scale (SES) by incorporating all the three dimensions of C-IRI used in Study 1 (EC, PT, and PD). We developed two questions for each of the three dimensions of C-IRI: two items assessed state empathic concern (SEC), “To what extent did you feel concerned about Heinz’s wife?” and “To what extent did you feel compassion for Heinz and his wife?” Two items assessed state personal distress (SPD), “To what extent did you feel distressed by the Heinz’s situation?” and “To what extent did you feel upset by Heinz’s situation?” Two items assessed state perspective taking (SPT), “To what extent did you try to imagine how you would feel in the Heinz’s place?” and “To what extent did you try to imagine things from Heinz’s perspective?” All items were assessed on 7-point Likert scales. The Cronbach’s alphas of the subscales were .79 (SES), .73 (SCE), .73 (SED), and .50 (SPD). There was no negative inter-correlation between SEC, SPD, and SPT subscales of the SES. Thus following previous studies [43,45], the total scores for these three subscales was computed as an indicator of state empathy.

### Results

#### Manipulation check

A manipulation check was conducted to ascertain whether participants in the experimental and control groups were similar in terms of trait empathy. Our analysis showed that participants in the pain group (*M* = 47.13, *SD* = 3.46) and control group (*M* = 46.53, *SD* = 3.95) were not significantly different in trait empathy, *t* (118) = 0.89, *p* > .05. We also checked the effectiveness of the physical pain manipulation, and results showed that participants’ pain ratings in the pain group (*M* = 5.82, *SD* = 1.62) was significantly higher than that in the control group (*M* = 1.05, *SD* = 1.31), *t* (118) = 17.31, *p* < .001.

#### Hypothesis testing

**Fig 3:**
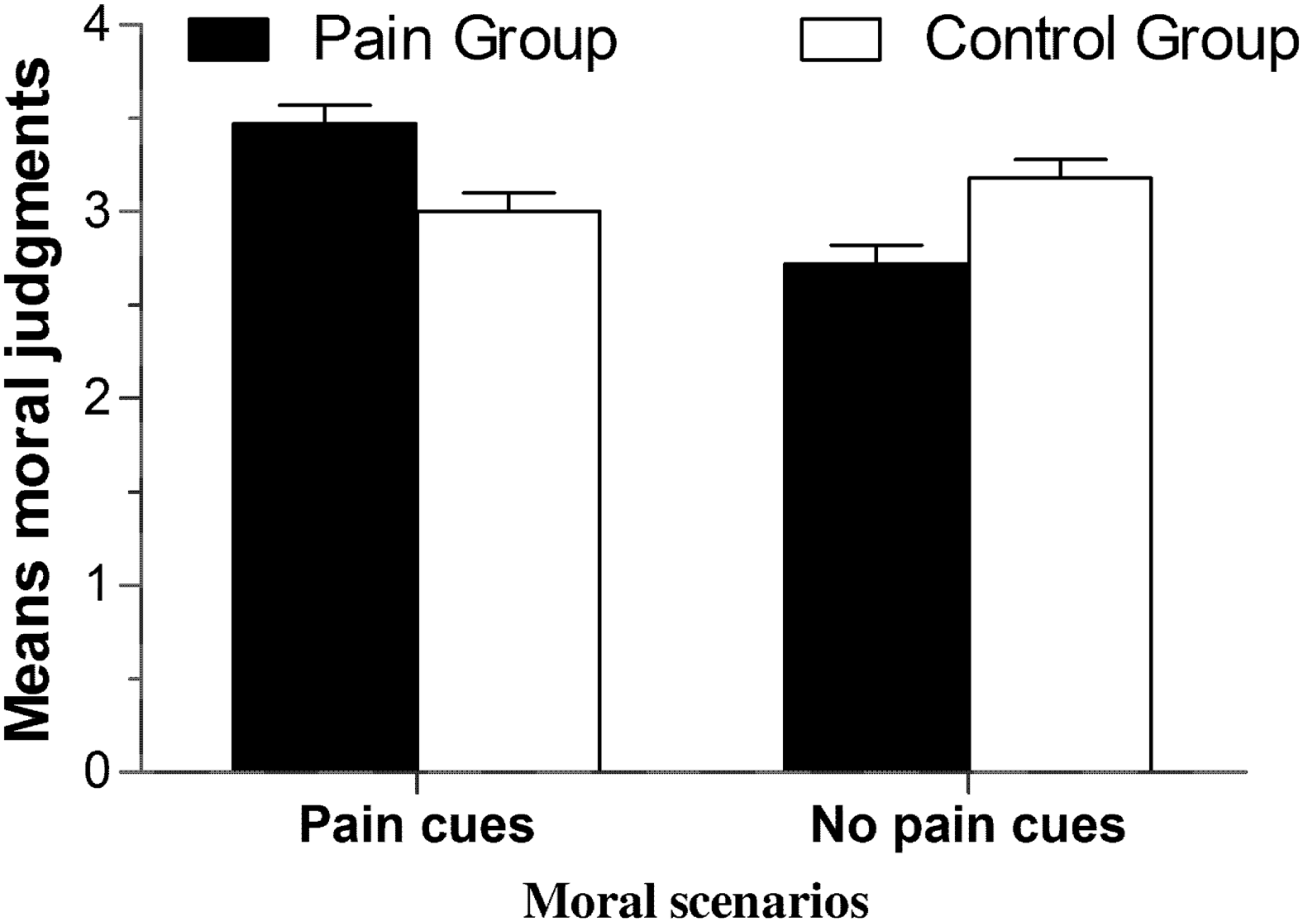
The bar graph of moral judgment in study two. This bar graph provides comparative view of the moral judgment across experimental conditions.

Descriptive statistics and correlation analysis results are shown in Table 3. An independent sample *t*-test showed that SES in the pain group (*M* = 29.23, *SD* = 5.34) was significantly higher than that in the control group (*M* = 25.98, *SD* = 5.51), *t* (118) = 3.28, *p* < .05, indicating that feeling physical pain enhanced participants’ self-assessments of state empathy. An analysis of variance (ANOVA) on moral judgment revealed a main effect of pain on moral judgment, *F* (1, 116) = 27.87, *p* < .001, *η*_p_^2^ = .19, suggesting that participants in the pain condition were more tolerant of Heinz’s behavior than those in the no pain condition (see Fig 3). However, we did not observe a significant main effect of moral scenario, *F* (1, 116) = 1.16, *p* > .05, *η*_p_^2^ = .01, nor did we observe a significant interaction effect between pain manipulation and moral scenario, *F* (1, 116) = 3.09, *p* > .05, *η*_p_^2^ = .03.

**Table 3:** Summary of descriptive statistics and correlation analysis results.

	*M*	*SD*	1	2	3	4	5	6	7	8
1.Moral judgment	3.09	.87	1							
2.SEC (State empathic concern)	9.53	2.52	.062	1						
3. SPT (State perspective taking)	9.32	2.21	.238**	.432**	1					
4.SED (State personal distress)	8.76	3.66	.064	.660**	.329**	1				
5. SES (Total scores of the state empathy scale)	27.61	5.64	.148	.877*	.721**	.818**	1			
6. Physical pain	3.43	2.81	.472*	.245*	.238**	.259**	.306**	1		
7. Affective empathy (AE)	25.53	2.32	-.151	.041	-.088	.156	.044	-.031	1	
8. Cognition empathy (CE)	21.33	2.49	-.022	-.050	.057	-.042	-.016	.003	.197*	1
9.Basic empathy (total scores of the basic empathy scale)	46.83	3.71	-.118	-.009	-.011	.076	.021	-.007	.758**	.783**

*. Correlation is significant at the 0.05 level (2-tailed).

**. Correlation is significant at the 0.01 level (2-tailed).

NOTE. N = 120.

**Fig 4:**
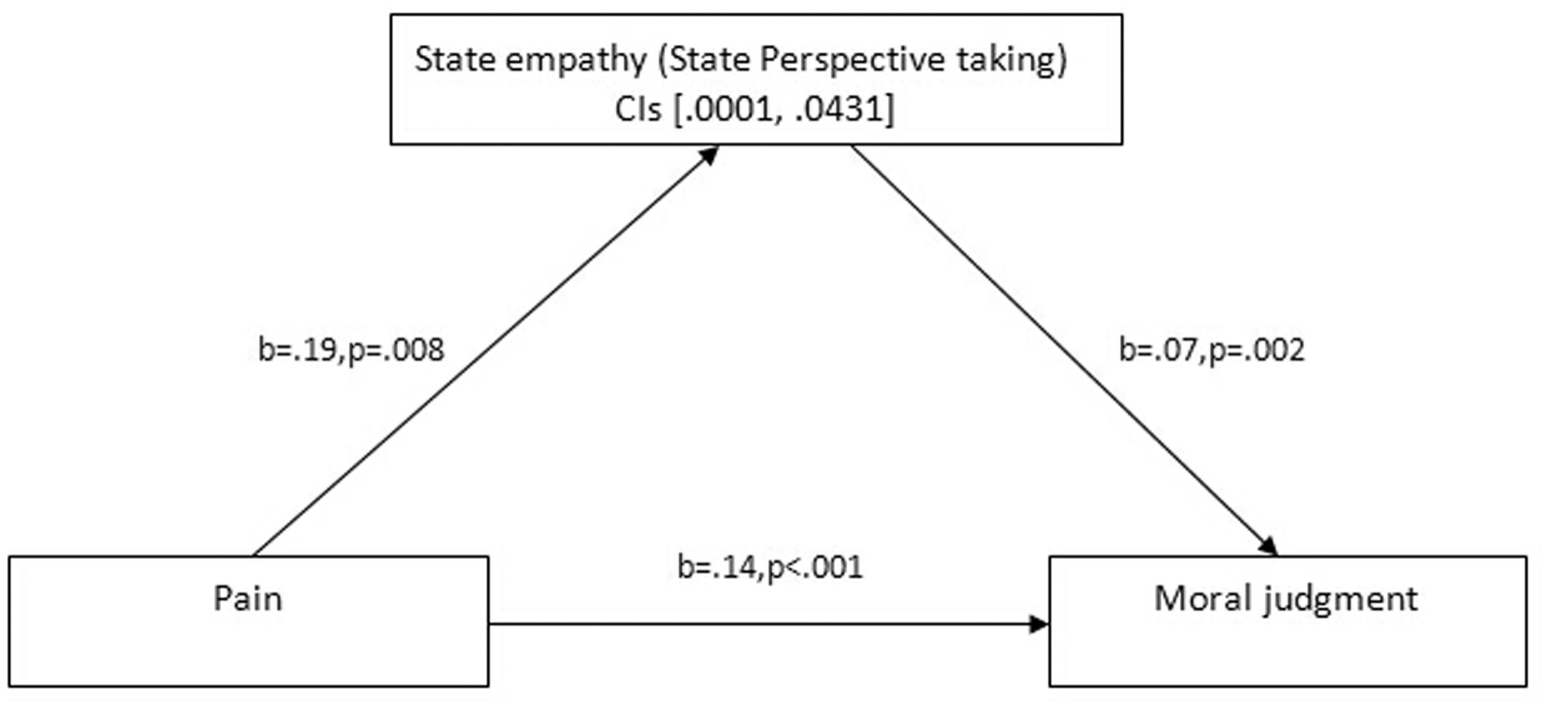
The mediation path of the effect of physical pain on moral judgment. Path model shows that the effect of physical pain on moral judgment is partially mediated by the state perspective taking subscale of the state empathy. Bias-corrected and accelerated 95% confidence intervals (CIS) from 5,000 bootstrap samples are reported for the indirect effect.

In addition, following the procedures outlined by Preacher and Hayes [46], we conducted mediation analyses with SES and the three subscale scores (SEC, SPD, and SPT) as mediators. The results showed that only the SPT score significantly mediated pain and moral judgment. Pain had a statistically significant effect on SPT (*b* = .19, SE = .007, *p* = .008), which, in turn, significantly affected moral judgment (*b* = .07, SE = .03, *p* = .001). The bootstrap analysis (with 5,000 iterations) showed that the 95% bias-corrected confidence interval for the size of the indirect effect excluded zero [.0001, .0431]. The direct effect of physical pain on moral judgment was non-significantly reduced (*b* = .14, SE = .02, *p* < .001) (see Fig 4).

## Discussion

Many studies have demonstrated that bodily states affect social cognition [50,51], including moral judgments [52,53], and a voluminous studies have addressed the relationship between pain and empathy [13,54], but few studies have focused on the influence of pain and pain-evoked empathy on moral judgments. Crockett and colleagues interestingly showed that participants who suffered painful electric shock evaluated others’ pain more intense than their own pain and they paid more money to reduce others’ pain [55]. The present study further explored how experiencing physical pain influences people’s moral judgments. We showed that experiencing physical pain induced participants to be more sympathetic in their moral judgments. Our findings provided support for embodied view of morality and Bastian and colleagues’ positive-functional view of pain [5].

Consistent with the “shared representation” and the “embodied simulation” accounts of the pain-empathy relationship, results from our two studies indicated that personal pain may facilitate people’s understanding of another individual’s pain or suffering. Thus, the perception of personal pain could be an important factor influencing people’s interpersonal interactions [56], because feeling pain might prepare an individual to take others’ perspectives and feel their experiences, thus affecting people’s judgment about their conduct. The correlational analyses showed that the PT subscale of the C-IRI (in study 1) and the SPT of the SES (in study 2) were significantly correlated with both feelings of physical pain and moral judgments (see Tables 1 and 2). Moreover, Study 2 also showed that the SPT significantly mediated the relationship between pain and moral judgment. These results were consistent with previous studies suggesting that perspective-taking can evoke helping behavior [57–59], and moral judgment [60,61].

Our analysis in Study 2 did not indicate that SES significantly mediated the relationship between physical pain and moral judgment, but only indicated the significant mediating role of SPT subscale. In certain ways, the incongruity between the two studies in our research is not surprising, since the C-IRI subscales used in Study 1 measures trait empathy, whereas the SES subscales used in Study 2 measures state empathy (i.e., empathetic reaction to situational cues). The state empathy measure is designed to capture situation-evoked empathic responses and therefore, it could be more variable across situations and measures [62] and more accurate in predicting other psychological phenomena in specific situations [49]. Situational cues might invoke cognitive and emotional systems of empathy that are underpinned by specific neural systems [63,64]. Researches have demonstrated that the experience of pain enables people to imagine the feelings of a person experiencing the same pain stimulus (i.e., cognitive dimension of empathy) [65,66], but not the emotional dimension of empathy [66]. In line with this finding, the results of Study 2 showed that the SPT (cognitive dimension of empathy), but not SPD, or SEC (emotional dimension of empathy), significantly mediated the relationship between pain and moral judgment. The results of Study 2 suggested that each dimension of empathy measures is sensitive to different situational cues. It is suggested that further researches is needed to more precisely identify the contextual determinants of empathy [67].

Our findings do not imply that participants’ empathic caring invoked by physical pain was the result of attentional bias towards specific pain cues. The fact that the main effect of moral scenarios (pain cue vs. no pain cue) on moral judgment is not significant in both studies indicates that participants’ empathic caring was the product of their processing of information about the whole situations involved in the moral scenarios.

Certain researchers have suggested that the influences of people’s first-hand physical pain on social interactions might have to do with their self-centered perspective. For instance, Mancini et al. [68] showed that in the Ultimatum Game (UG), proposers who suffered laser pain decreased fair offers, whereas suffering responders increased their acceptance rate. The authors suggested that the personal experience of pain facilitates the emergence of a self-centered perspective that guides participants to maximize self-gain. Although this explanation sounds reasonable, one could also refer to literature on the psychology of power when considering the roles of responders and the proposers in the UG. The payoffs of the responders were largely determined by the proposers and therefore, such roles would evoke the feeling of powerfulness in the proposers and powerlessness in the responders [69]. It is known that powerfulness is associated with a reduced tendency to take other’s perspective. Therefore, introducing physical pain could lead the proposers to associate unpleasant feeling with the bargaining relationship, thus leading to more self-centered decision, such as an increase in unfair offers. Simultaneously, physical pain could reinforce the feeling of powerlessness in responders, leading to a more obedient decision strategy when facing unfair offers, such as the increased acceptance of unfair offers. This assumption suggests that the effects of physical pain on human judgment and decision making are contingent on experimental settings. Clearly, the experimental design in Mancini et al. is different from that of the current study in that there were no monetary incentives and no role-induced feelings of power in this research. Therefore, findings of Mancini et al. do not necessarily contradict the findings of this study. It is suggested that future research should be designed to identify the moderating roles of contextual factors (e.g., participants’ roles) in the effects of physical pain on people’s judgment and decision making.

Finally, study 1 shows that empathy partially mediated the effects of painful feelings on moral judgment, there could be other factors implicated in this relationship. Group affiliation could reasonably exert its influence in shaping participants’ moral judgment after experiencing physical pain. In accordance with the positive function of pain proposed by Bastian et al. [5], pain experience can improve affiliation with others, and such motivation can be explained by the “tend-and-befriend hypothesis” [70]. According to this view, motivation to affiliate with others under conditions of stress is a common response in humans, because the affiliation motive under such negative situations as experiencing pain appears to constitute an evolutionarily stable instinct. Future studies could obtain clearer evidence regarding the determining role of empathy or a group affiliation motive in shaping people’s moral judgments when they are experiencing pain.

## Conclusion

In summary, the results of two studies showed that ice-induced physical pain can induce a person to be more sympathetic in a moral judgment, and that self-assessment of trait empathy (in study 1) and state perspective taking of State Empathy Scale (in study 2) mediated the effects of painful feelings on moral judgment. Our findings provide support for embodied view of morality and the view that pain can serve a positive psychosocial function.

## Supporting Information

S1 Dataset The original data of the study one. (SAV)

S2 Dataset The original data of the study two. (SAV)

## References

[1] Merksey H, Bogduk N (1994) Pain terms: a current list with definitions and notes on usage. IASP Task Force on Taxonomy, Merksey H, Bogduk N, ed. Classification of chronic pain: descriptions of chronic pain syndromes and definitions of pain terms. 2nd edition Seattle: IASP Press.

[2] Decety J, Ickes W (2011) The social neuroscience of empathy: MIT Press.

[3] Bastian B, Jetten J, Ferris L (2014) Pain as social glue: shared pain increases cooperation. Psychol sci 25: 2079–2085. 10.1177/0956797614545886 25193943

[4] Alessandra M, Viviana B, Maria Serena P, Enea Francesco P, Salvatore Maria A (2011) Suffering makes you egoist: acute pain increases acceptance rates and reduces fairness during a bilateral ultimatum game. Plos One 6: e26008 10.1371/journal.pone.0026008 22022492 PMC3192138

[5] Bastian B, Jetten J, Hornsey MJ, Leknes S (2014) The positive consequences of pain: a biopsychosocial approach. Pers Soc Psychol Rev 18: 256–279. 24727972 10.1177/1088868314527831

[6] Pavey L, Greitemeyer T, Sparks P (2012) “I help because I want to, not because you tell me to”. Pers Soc Psychol Bull 38: 681–689. 10.1177/0146167211435940 22326945

[7] Piliavin JA, Charng H-W (1990) Altruism: A review of recent theory and research. Ann rev soc: 27–65.

[8] Batson CD (2011) Altruism in humans: Oxford University Press.

[9] Dovidio JF, Piliavin JA, Schroeder DA, Penner L (2006) The social psychology of prosocial behavior: Lawrence Erlbaum Associates Publishers.

[10] Jackson PL, Rainville P, Decety J (2006) To what extent do we share the pain of others? Insight from the neural bases of pain empathy. Pain 125: 5–9. 16997470 10.1016/j.pain.2006.09.013

[11] Lamm C, Decety J, Singer T (2011) Meta-analytic evidence for common and distinct neural networks associated with directly experienced pain and empathy for pain. Neuroimage 54: 2492–2502. 10.1016/j.neuroimage.2010.10.014 20946964

[12] Lawrence E, Shaw P, Giampietro V, Surguladze S, Brammer M, David A (2006) The role of ‘shared representations’ in social perception and empathy: an fMRI study. Neuroimage 29: 1173–1184. 16337816 10.1016/j.neuroimage.2005.09.001

[13] Singer T, Seymour B, O'Doherty J, Kaube H, Dolan RJ, Frith CD (2004) Empathy for pain involves the affective but not sensory components of pain. Science 303: 1157–1162. 14976305 10.1126/science.1093535

[14] Decety J, Cowell JM (2015) Empathy, Justice, and moral behavior. AJOB Neurosci 6: 3–14. 26877887 10.1080/21507740.2015.1047055 PMC4748844

[15] Benuzzi F, Lui F, D, Nichelli P, Porro C (2008) Does it look painful or disgusting? Ask your parietal and cingulate cortex. J Neurosci 28: 923–931. 10.1523/JNEUROSCI.4012-07.2008 18216200 PMC6670998

[16] Costantini M, Galati G, Romani GL, Aglioti SM (2008) Empathic neural reactivity to noxious stimuli delivered to body parts and non-corporeal objects. Eur J Neurosci 28: 1222–1230. 10.1111/j.1460-9568.2008.06406.x 18783380

[17] Cheng Y, Yang CY, Lin CP, Lee PL, Decety J (2008) The perception of pain in others suppresses somatosensory oscillations: a magnetoencephalography study. Neuroimage 40: 1833–1840. 10.1016/j.neuroimage.2008.01.064 18353686

[18] Whitmarsh S (2011) Sensorimotor alpha activity is modulated in response to the observation of pain in others. Front Hum Neurosci 5: 91 10.3389/fnhum.2011.00091 22007165 PMC3188815

[19] Riečanský I, Paul N, Kölble S, Stieger S, Lamm C (2014) Beta oscillations reveal ethnicity ingroup bias in sensorimotor resonance to pain of others. Soc Cogn Affect Neurosci 10: 893–901. 10.1093/scan/nsu139 25344947 PMC4483561

[20] Avenanti A, Bueti D, Galati G, Aglioti SM (2005) Transcranial magnetic stimulation highlights the sensorimotor side of empathy for pain. Nat Neurosci 8: 955–960. 15937484 10.1038/nn1481

[21] Avenanti A, Paluello IM, Bufalari I, Aglioti SM (2006) Stimulus-driven modulation of motor-evoked potentials during observation of others' pain. Neuroimage 32: 316–324. 16675270 10.1016/j.neuroimage.2006.03.010

[22] Aziz-Zadeh L, Sheng T, Liew SL, Damasio H (2012) Understanding otherness: the neural bases of action comprehension and pain empathy in a congenital amputee. Cerebral Cortex 22: 811–819. 10.1093/cercor/bhr139 21734252 PMC6276973

[23] Gallese V (2007) Before and below 'Theory of Mind': Embodied Simulation and the Neural Correlates of Social Cognition. Philos Trans R Soc Lond B: Biol Sci 362: 659–669. 17301027 10.1098/rstb.2006.2002 PMC2346524

[24] Goldman A, de Vignemont F (2009) Is social cognition embodied? Trends Cogn Sci 13: 154–159. 10.1016/j.tics.2009.01.007 19269881

[25] Preston SD, Waal FBMD (2002) Empathy: Its ultimate and proximate bases. Behav Brain Sci 25: 1–20; discussion 20–71. 12625087 10.1017/s0140525x02000018

[26] Gallese V, Keysers C, Rizzolatti G (2004) A unifying view of the basis of social cognition. Trends Cogn Sci 8: 396–403. 15350240 10.1016/j.tics.2004.07.002

[27] De VF, Singer T (2006) The empathic brain: how, when and why? Trends in Cognitive Sciences 10: 435–441. 16949331 10.1016/j.tics.2006.08.008

[28] Lamm C, Batson CD, Decety J The Neural Substrate of Human Empathy: Effects of Perspective-taking and Cognitive Appraisal. J Cogn Neurosci 19: 42–58. 17214562 10.1162/jocn.2007.19.1.42

[29] Jackson PL, Meltzoff AN, Decety J (2005) How do we perceive the pain of others? A window into the neural processes involved in empathy. Neuroimage 24: 771–779. 15652312 10.1016/j.neuroimage.2004.09.006

[30] Saarela MV, Hlushchuk Y, Williams ACdC, Schürmann M, Kalso E, Hari R (2007) The Compassionate Brain: Humans Detect Intensity of Pain from Another's Face. Cerebral Cortex 17: 230–237. 16495434 10.1093/cercor/bhj141

[31] Nadia B, Angela R, Silvia C, Giuseppe V (2013) Understanding others' feelings: the role of the right primary somatosensory cortex in encoding the affective valence of others' touch. J Soc Neurosci 33: 4201–4205. 10.1523/JNEUROSCI.4498-12.2013 PMC6619319 23447627

[32] Decety PRJ (2004) How would you feel versus how do you think she would feel?A neuroimaging study of perspective-taking with social emotions. J Cogn Neurosci 16: 988–999. 15298786 10.1162/0898929041502661

[33] Bastiaansen JA, Thioux M, Keysers C (2009) Evidence for mirror systems in emotions. Philos Trans R Soc Lond B: Biol Sci 364: 2391–2404. 19620110 10.1098/rstb.2009.0058 PMC2865077

[34] Bastian B, Jetten J, Fasoli F (2011) Cleansing the soul by hurting the flesh: The guilt-reducing effect of pain. Psychol Sci 22: 334–335. 10.1177/0956797610397058 21245493

[35] Scott J, Huskisson E (1976) Graphic representation of pain. Pain 2: 175–184. 1026900

[36] Downie W, Leatham P, Rhind V, Wright V, Branco J, Anderson J (1978) Studies with pain rating scales. Ann Rheum Dis 37: 378–381. 686873 10.1136/ard.37.4.378 PMC1000250

[37] Williamson A, Hoggart B (2005) Pain: a review of three commonly used pain rating scales. J Clin Nurs 14: 798–804. 16000093 10.1111/j.1365-2702.2005.01121.x

[38] Lin Q, Xue Z, Yanfei W (2009) Revision of the positive affect and negative affect scale. CHN J Appl Psychol 14: 249–254.

[39] Watson D, Clark LA, Tellegen A (1988) Development and validation of brief measures of positive and negative affect: the PANAS scales. J Pers Soc Psychol 54: 1063–1070. 3397865 10.1037//0022-3514.54.6.1063

[40] Rong X, Sun BH, Huang XZ, Cai MY, Li WJ (2010) Reliabilities and validities of Chinese version of Interpersonal Reactivity Index. CHN J Clin Psycho: 158–160.

[41] Davis MH (1983) Measuring individual differences in empathy: evidence for a multidimensional approach. J Pers Soc Psychol 44: 113–126.

[42] Trobst KK, Collins RL, Embree JM (1994) The role of emotion in social support provision: Gender, empathy and expressions of distress. J Soc Pers Relat 11: 45–62.

[43] Burke DM (2001) Empathy in sexually offending and nonoffending adolescent males. J Interpers Violence 16: 222–233.

[44] Moriarty N, Stough C, Tidmarsh P, Eger D, Dennison S (2001) Deficits in emotional intelligence underlying adolescent sex offending. J Adolescence 24: 743–751. 10.1006/jado.2001.0441 11790054

[45] Gini G, Albiero P, Benelli B, Altoè G (2007) Does empathy predict adolescents' bullying and defending behavior? Aggressive Behavior 33: 467–476. 17683107 10.1002/ab.20204

[46] Preacher KJ, Hayes AF (2008) Asymptotic and resampling strategies for assessing and comparing indirect effects in multiple mediator models. Behav Res Methods 40: 879–891. 18697684 10.3758/brm.40.3.879

[47] Xia D (2011) Study on reliability and validity and preliminary application of the basic empathy scale. Zheng zhou University 1–71 p.

[48] Jolliffe D, Farrington DP (2006) Development and validation of the basic empathy scale. J Adolescence 29: 589–611. 10.1016/j.adolescence.2005.08.010 16198409

[49] Fehr R, Gelfand MJ, Nag M (2010) The road to forgiveness: a meta-analytic synthesis of its situational and dispositional correlates. Psychological Bulletin 136: 894–914. 10.1037/a0019993 20804242

[50] Jostmann NB, Lakens D, Schubert TW (2009) Weight as an embodiment of importance. Psychol Sci 20: 1169–1174. 10.1111/j.1467-9280.2009.02426.x 19686292

[51] Ackerman JM, Nocera CC, Bargh JA (2010) Incidental haptic sensations influence social judgments and decisions. Science 328: 1712–1715. 10.1126/science.1189993 20576894 PMC3005631

[52] Schnall S, Haidt J, Clore GL, Jordan AH (2008) Disgust as embodied moral judgment. Pers Soc Psychol Bull 34: 1096–1109. 10.1177/0146167208317771 18505801 PMC2562923

[53] Zhong C-B, Strejcek B, Sivanathan N (2010) A clean self can render harsh moral judgment. J Exp Soc Pyschol 46: 859–862.

[54] Goubert L, Craig KD, Vervoort T, Morley S, Sullivan M, Williams ACC, et al (2005) Facing others in pain: the effects of empathy. Pain 118: 285–288. 16289804 10.1016/j.pain.2005.10.025

[55] Crockett MJ, Kurth-Nelson Z, Siegel JZ, Dayan P, Dolan RJ (2014) Harm to others outweighs harm to self in moral decision making. Proc Natl Acad Sci 111: 17320–17325. 10.1073/pnas.1408988111 25404350 PMC4260587

[56] Valeriani M, Betti V, Le Pera D, De Armas L, Miliucci R, Restuccia D, et al (2008) Seeing the pain of others while being in pain: a laser-evoked potentials study. Neuroimage 40: 1419–1428. 10.1016/j.neuroimage.2007.12.056 18291679

[57] Eisenberg N, Miller PA (1987) The relation of empathy to prosocial and related behaviors. Psychological Bulletin 101–119. 3562705

[58] Batson CD, Early S, Salvarani G (1997) Perspective taking: Imagining how another feels versus imaging how you would feel. Pers Soc Psychol B 23: 751–758.

[59] Cialdini RB, Brown SL, Lewis BP, Luce C, Neuberg SL (1997) Reinterpreting the empathy–altruism relationship: When one into one equals oneness. J Pers Soc Psychol 73: 481–494. 9294898

[60] Mason MG, Gibbs JC (1993) Social perspective taking and moral judgment among college students. J Adolescent Res 8: 109–123.

[61] Eisenberg N (2010) Empathy-related responding: Links with self-regulation, moral judgment, and moral behavior In Mikulincer M & Shaver P R (Eds), Prosocial motives, emotions, and behavior: The better angels of our nature (pp 129–148) Washington, DC: American Psychological Association.

[62] Reynolds WJ, Presly AS (1988) A study of empathy in student nurses. Nurse Educ Today 8: 123–130. 3419399 10.1016/0260-6917(88)90029-9

[63] Decety J, Jackson PL (2004) The functional architecture of human empathy. Behav Cogn Neurosci Rev 3: 71–100. 15537986 10.1177/1534582304267187

[64] Shamay-Tsoory SG, Aharon-Peretz J, Perry D (2009) Two systems for empathy: a double dissociation between emotional and cognitive empathy in inferior frontal gyrus versus ventromedial prefrontal lesions. Brain 132: 617–627. 10.1093/brain/awn279 18971202

[65] Batson CD, Sympson SC, Hindman JL, Decruz P, Todd RM, Weeks JL, et al (1996) " I've been there, too": Effect on empathy of prior experience with a need. Pers Soc Psychol B 22: 474–482.

[66] Preis M, Kroener‐Herwig B (2012) Empathy for pain: The effects of prior experience and sex. European journal of pain 16: 1311–1319. 10.1002/j.1532-2149.2012.00119.x 22949180

[67] Singer T, Lamm C (2009) The social neuroscience of empathy. Annals Of The New York Academy Of Sciences 1156: 81–96. 10.1111/j.1749-6632.2009.04418.x 19338504

[68] Mancini A, Betti V, Panasiti MS, Pavone EF, Aglioti SM (2011) Suffering makes you egoist: acute pain increases acceptance rates and reduces fairness during a bilateral ultimatum game. PloS one 6: e26008 10.1371/journal.pone.0026008 22022492 PMC3192138

[69] Galinsky AD, Magee JC, Inesi ME, Gruenfeld DH (2006) Power and perspectives not taken. Psychol sci 17: 1068–1074. 17201789 10.1111/j.1467-9280.2006.01824.x

[70] Taylor SE, Gonzaga GC, Klein LC, Hu P (2006) Relation of oxytocin to psychological stress responses and hypothalamic-pituitary-adrenocortical axis activity in older women. Psychosomatic Medicine 68: 238–245. 16554389 10.1097/01.psy.0000203242.95990.74

